# 

**Published:** 1888-03-24

**Authors:** 


**SPECIAL NOTICE TO NEWSAGENTS, ADVERTISERS, AND THE TRADE.**

**CHANGE OF OFFICE AND PUBLISHER.**

Notice is hereby given that the Office of The Hospital will hence forth be at 38, Old Jewry (Cheapside end), E.C., to which address all communications should be sent. Mr. A. Doig has been appointed Advertising Agent, and Mr. J. H. S. Hanning, Manager. All Cheques and Post Office Orders should be made payable to J. H. S. Hanning, 38, Old Jewry, E.C. crossed Lloyds, Barnetts, and Bosanquets Bank (Limited).

Next week being Easter week. The Hospital will be published on Wednesday, the 28th, instead of Thursday.

**TO SUPERINTENDENTS, SECRETARIES, AND MATRONS.**

The Editor will be glad to have a copy of the Regulations, and also of every Report issued from the Press of Asylums, Hospitals, Convalescent and Nursing Institutions, as well as those of other Charities, so soon as they are published, and an early copy in duplicate of all appeals for funds. Notices of Annual and other Meetings, Festival Dinners, etc., will be welcomed All such communications should be addressed to *The Editor, The Lodge, Porchester Square, London, W.* Any superintendent secretary, matron, or other chief official who will regularly send such documents and information will be registered to receive free of cost a cover for binding The Hospital in half-yearly volumes on sending name and address to the publisher.

### Amusements with Profit for all Ages.

**Rules.**—All answers must be addressed to the Prize Editor, The Hospital, 38, Old Jewry, Cheapside, London, E.C., not later than the *first* post on Thursday next. The full name and address must in all cases be given, but a *nom de plume* may be added for publication. Only one side of the paper must be written on.

### FOR MEN AND WOMEN.

**Rule.**—Competitors can enter for *all* quarterly competitions, but no competitor may take more than *two quarterly prizes* during the year.

### Acrostic Competition.

Second quarter commenced February 25th, 1888; ends May 19th, 1888.

A PRIZE of ONE GUINEA will be given to the competitor who gains the highest number of marks for best original acrostics during the ensuing quarter. All acrostics sent in for this competition will be credited according to merit, twelve marks being the highest score given for one acrostic.

A PRIZE of HALF-A-GUINEA to be given to the competitor who gains the highest score for solution of acrostics set. *One mark* only will be given to the competitors who solve all the lights of each acrostic.

Exception will be made in the case of *quotation acrostics*, when *two marks* will be given, *one* for the correct solution of all *lights*, and *one* mark for the *source* of the quotations.

This week credit will be given for

No. 3. Double Acrostic.

A man of peace.
"God s finger touched him, and he slept."
Die and enrich a college or a cat."

2. ' How much thy years excel
In marts of council, and in speaking well."

3. "That rarest tuner,
Henceforth in April,
Shall wake the sooner."

4. "Each awful curse that on this mountain rang,
Peals with a direr clang,
Out of that silver trump, whose tones of old,
Forgiveness only told."

5. "While the tree of freedom's withered trunk puts forth a leaf,
Ever for thy tomb a garland let it be."

6. "O rose of May!
Dear maid, kind sister."

7. "Doing good only wherever it spreads,
Not evil."

### Answers.

No. 2. Double Acrostic.

R yth M

E ch O

P e N

U mbrell A

B alde R

L i'a C

I nc H

C harit Y

Correct answers have been received from Ostrich, Rowdy Corner, Brownie, Tabs, Mad Hatter, Dunce, E. E., Gretta, Mater, Reldas, Lady Clara.

### (A) THE WORD COMPETITION.

Second Quarterly Competition commenced February 4th, 1888; ends April 28th, 1888.

Three prizes of one guinea, 15*s.* and 10*s.* 6*d.* will be given each quarter to the three competitors who obtain the highest number of marks; (1) by making the largest number of words derived from the word or phrase set for anatomical dissection; or (2) for the best answers to (B); or (3) by (1) and (2) combined.

We shall give the newspapers and periodicals for dissection during this quarter, that for this, the *eighth* week of the quarter, being

"Church Times."

Observe.—Proper names, abbreviations, foreign words, words of less than four letters, and repetitions are barred; plurals, and past and present participles, of verbs are allowed. Nuttall's *Standard* d ictionary only to be used.

N.B.—All words sent in must be arranged *alphabetically*, with correct total affixed.

### Results of Sixth Week.

*Guardian.*—Lodge (43), Sym (43), Patience (41), Gretta (41), Ellice (40), E. E. (40), Butterfly (39), Lauriston 38), Lancashire Witch (38), Lightowlers 37), Brisbane (37), Dollie (35), Nelle (34), Dunce (31).

### (B) THE LITERARY COMPETITION.

Some people hate puzzles of all kinds, including word competitions, but they are fond of correspondence, and many sisters and mothers are continually receiving and sending letters to and from members of the family who are abroad in India or the Colonies. The constant coming and going between England and other portions of the British Empire make it a matter of interest and importance to know something about the life, the climate, the amusements, the adventures, the clothing, and the surroundings of those who seek a livelihood beyond the seas. We shall therefore try to find space for the publication of selected letters from abroad, which may be sent by any of our readers who have friends and relations in foreign lands. We shall give marks for these letters as well as for the word competitions which will count equally for these prizes, so that anyone may enter for both or either, the prizes to be given to those who get the highest number of marks.

---

### DOUBLE ACROSTIC.

NazaretH.
'Tis hence we learn how toil that common seem'd
Is to nobility by love redeemed.

UnitE.
Alone, thou canst not: in alliance,
Ye can to failure bid defiance!

ReligioN.
French hospitals, where sick men die,
This one sure medicine deny.

SisteR.
So pain to tenderness, unchid, appeals,
For each the brotherhood of suffering feels.

EcstasY.
Rapt out of self, my spirit's gaze
Delighted threads æthereal ways.

SeraphiC.
No doctor he of this corporeal frame
Who won this epithet to adorn his name.

PuB.
Slang: yes! but name too deeply scored
On those who throng the "Drunken Ward!"

EaU.
French: yes! but, oh, how much abhorred
By those who fill the "Drunken Ward!"

NeighbouR.
Bind up his wounds: pour in thy oil and wine,
And Heaven's high recompense, dear soul, is thine!

SaveD!
Fire! death in front, and death behind:
One leap! and life! for God is kind.

IcE.
"When pain and anguish wring the brow,
A ministering angel thou."

OculisT.
Man's darkness to enlighten! Blessed art!
Of the True Light's own ministry a part.

NeglecT.
Oh shame! if you for whom I pen these rhymes
Incur this guiltiest of all human crimes.

---

The project, which, with sympathetic art
Your clever brains suggest,
Surely, O friend! a nation's generous heart
Responsively has blest!

Pasteur.

### THE CHILDREN'S CORNER.

PRIZES.
FOR MOST CORRECT ANSWERS TO PUZZLES.

This Commenced October 1st, 1887.

One Pound a Month.

A PRIZE OF THREE GUINEAS to the Competitor who shall have sent in the largest number of correct or best answers during the twelve months.

### Puzzles—Fourth Week.

No. 13. Arithmetical Puzzle.—Two friends have incomes of precisely equal amount. George saves one-fifth of his income, while James spends £80 a-year more than his friend, and at the end of four years finds himself £220 in debt Required the income and expenditure of each.

No. 14. Double Acrostic.—1. A plant found in waste places. 2. A species of wild sheep found in Siberia, etc. 3. Scotch name for a tree bearing lovely red berries. 4. Canary's salad. 5. River horse of Africa. Initials and finals form the name of a month in the year, and something by which it is characterised.

No. 15. Enigma.
Adam my parent was, 'tis very true;
And, what's more strange, I always am with you.
With insects, birds, and beasts—in short, what not?
'Tis I distinguish truly what is what.

No. 16. Diamond Puzzle. 1. A consonant 2. An English Bishop. 3. An English musical composer. 4. A great scholar of the 18th century. 5. A celebrated musical composer. 6. A marine monster. 7. An Arctic Explorer. 8. The inventor of a loom. 9. A consonant.

```
    .
   . . .
  . . . . .
 . . . . . . .
. . . . . . . . .
 . . . . . . .
  . . . . .
   . . .
    .
```

### Results of Second Week Puzzles.

No. 5. Riddle-me-ree. Daisy.
No. 6. Historical Acrostic.

R hubar B
I dy L
C — O
H or N
A nchore D
R o E
D rol L

No. 7. Charade.—Butter-fly=Butterfly.
No. 8. Literary Conundrum. "Gay," "Young," "Lamb."

*All right*—Jumps, Squeaker. *Three right*—Bagshot, Alsie, Bookworm, Umslopogaas, A. C. K., *Two right*—A. G. S., McCulloch, Plummy Fluff, Scrooge, Happy Eliza, Spider, Mount Edgecumbe, Babette.

### Notices to Correspondents.

*Excelsior*—Puzzles received too late.

---

### Conditions.

I. Every paper sent in must be clearly written, and one side only must be used. All competitors must mark at the head of their papers the number of their competition.

II. Each paper must be signed by the author with his or her real name and address. A *nom de plume* may be added, if the writer does not desire to be referred to by us by his real name. In the case of all prize-winners, however, the real name and address will be published.

III. All communications relating to prize competitions should be addressed to "The Prize Editor, The Hospital, 38, Old Jewry, London, E.C." Competitors are requested not to include in letters, etc., to the Prize Editor communications on matters of other business. All letters must be fully prepaid.

IV. The Prize Editor of "The Hospital" is the sole judge of the competitions, and his decision is in all cases final and without appeal.

V. The Prize Editor reserves the right to publish any paper sent in, whether it receives a prize or not. He does not hold himself responsible for any MSS. sent, nor can he undertake to return rejected competitions.

VI.—All children resident at school are requested to forward their *home* address in addition to the school one, when entering for the "Children's Corner" Competitions.

**Notice to all Correspondents.**—N.B.—All letters referring to this page, which do not arrive at 38, Old Jewry, Cheapside, London, E.C., by the *first post on Thursdays*, and are not addressed PRIZE EDITOR, will in future be disqualified and disregarded.

No one will be permitted to win the Monthly or Quarterly Prizes twice in any one year, the Annual prizes being offered especially to prevent this.

